# Socioeconomic Status and Breast Cancer Treatment in the United States: Results From a Systematic Literature Review

**DOI:** 10.1177/10732748251341520

**Published:** 2025-05-09

**Authors:** Sreelatha Akkala, Mohammed Zuber, Julie Alaere Atta, Nompumelelo O. Mzizi, Jyothi Akkula

**Affiliations:** 1 School of Public Health, 12340 The University of Texas Health Science Center at Houston, Houston, TX, USA; 2 College of Pharmacy, University of Georgia, Athens, GA, USA; 3 Independent Researcher, Hyderabad, India

**Keywords:** socioeconomic status, health care disparities, breast cancer, treatment delay, access to care, breast cancer treatment disparities, social determinants of health

## Abstract

**Background:**

In 2024, breast cancer is the second most common cancer globally, affecting 2.3 million women. In the United States (US), 310 720 new female breast cancer cases were estimated in 2024. Existing research has identified substantial disparities in breast cancer treatment and survival based on socioeconomic status (SES). This systematic review examines the association between the key SES indicators income, education, and occupation on breast cancer outcomes among the US breast cancer patients.

**Methods:**

An electronic search was conducted using Medline®, Embase®, and Web of Science, from inception to December 2023. Observational studies examining the influence of SES indicators on breast cancer outcomes, including treatment receipt, adherence, and survival, were included. Data were summarized qualitatively due to heterogeneity in SES measures and outcome definitions.

**Results:**

Of 2600 studies retrieved from the searches, 23 studies met the inclusion criteria (19 cohort, 4 cross-sectional). Women with lower SES were less likely to receive or experience delay in receiving the recommended treatment than women with higher SES. In addition, women with low household income (<$25,000) were more likely to report discontinuations in therapy as compared to women with an income of $50,000 or more. Education level influenced treatment adherence and timely care, with higher educational attainment linked to improved survival rates. Occupational status impacted treatment continuity, with low-wage jobs and inflexible work schedules contributing to delays and discontinuation of care.

**Conclusion:**

SES significantly influences breast cancer care and survival, with lower SES associated with delayed treatment, poorer adherence, and worse outcomes. Healthcare interventions and policies focusing on equitable access to quality care tailored to all women, regardless of their socioeconomic background, may improve breast cancer outcomes for the patients with various demographic characteristics in the US.

## Introduction

Breast cancer is one of the most prevalent malignancies among women across the world, posing a significant public health challenge.^1^ In 2024, breast cancer ranks as the second most prevalent cancer in both the developed and the developing regions, affecting 2.3 million women annually, leading to an estimated 665, 684 deaths globally each year.^2^ In the United States (US), 310 720 new female breast cancer cases were estimated in 2024.^3^ Despite considerable advancements in screening, diagnosis, and treatments, disparities in breast cancer outcomes persists, primarily driven by socioeconomic status (SES), which influences both treatment-seeking behaviors and the quality of care received.^4^ These disparities underscore the complex interplay between SES factors and breast cancer outcomes, affecting all the stages of care continuum, from initial therapy to ongoing survivorship.^5^

SES is typically assessed through indicators such as income, education level, occupation, area of residence, insurance status, deprivation index.^6,7^ Lower SES is commonly associated with low healthcare utilization, low health literacy, and financial constraints which collectively delay access to quality care.^8^ High out-of-pockets costs can cause financial distress among patients, often compelling them to incur debt, deplete savings, or engage in cost-coping strategies, including nonadherence to prescribed treatment regimens.^9^ These barriers may lead to decreased survival rates and lower quality of life among breast cancer patients with lower socioeconomic backgrounds.^10^

Education, which is a part of SES index, is a strong determinant of breast cancer prognosis. Evidence suggests that patients with higher levels of education have better health literacy, which can positively influence patient’s understanding of the disease etiology and management strategies.^11^ Research indicates that women with higher educational attainment are more likely to participate in screening programs, adhere to the treatment plans, and seek high quality healthcare, contributing to increased survival rates.^12^ In contrast, individuals with lower education levels often present at later stages of the disease and have poor compliance with prescribed treatment regimens, resulting in a higher likelihood of recurrence and lower survival rates.^13^

Occupational status represents another SES factor with implications on breast cancer outcomes.^14^ People with low-wage employment may experience inflexible work schedules which can result in missed therapy sessions and inadequate post-operative care. Moreover, insurance coverage which is often linked to occupational status, varies significantly, with many low-status jobs providing minimal or no insurance benefits to cover required treatment.^14,15^ Furthermore, patients in physically demanding occupations often face challenges in completing treatment regimens, as workplace demands and lack of employer support contribute to interruptions in care.^16^

The cumulative impact of the key SES indicators income, education, and occupation results in substantial disparities in breast cancer treatment and long-term survival.^17^ Evidence consistently shows that patients with higher SES levels access more comprehensive treatments, experience better survival rates, lower recurrence rates, and improved quality of life compared to individuals with low SES backgrounds.^17^ Addressing these inequalities is essential for public health, necessitating targeted measures and policy reforms aimed at improving care for individuals of lower SES.^14^ This study aims to systematically review the existing evidence on SES and breast cancer outcomes, specifically examining the influence of SES factors on receipt of treatment, adherence, and survival in US patients. SES is a multidimensional construct that significantly influences health outcomes, including breast cancer treatment and survival. Given the extensive heterogeneity in SES measurement and its interplay with healthcare outcomes, our study focuses on three key SES indicators income, education, and occupation.^6,7^ These factors are widely recognized for their direct and measurable impact on disparities in breast cancer care. While other SES dimensions may also contribute to inequities, incorporating all potential indicators would substantially increase the complexity of this analysis. This review helps inform healthcare policymakers to address SES-driven disparities and approaches to mitigate inequalities in breast cancer incidence and outcomes among women.

## Methods

This systematic review followed the 2020 Preferred Reporting Items for Systematic Reviews and Meta‐Analyses (PRISMA) reporting guideline.^18^ This study’s protocol was registered in the International Prospective Register of Systematic Reviews (PROSPERO: CRD420251009394).

### Literature Search

A comprehensive search was performed in Ovid Medline®, EMBASE®, and Web of science databases from inception to December 2023. Keywords included “income,” “education,” “occupation,” “SES,” and “breast cancer,” with filters for human studies and no language restrictions. Abstracts from conferences of the American Society of Clinical Oncology (ASCO) and the American Association for Cancer Research (AACR) from 2020-2023 were hand-searched. We also searched grey literature and Google Scholar to include relevant studies.

### Study Selection and Inclusion/Exclusion Criteria

Our study included women aged 18 years older with Stage I-III breast cancer diagnosis. Stage IV breast cancer or other types of cancer, studies outside US, reviews, editorials, commentary, and studies not published in English were excluded. Observational studies (retrospective cohort, case-control, cross-sectional) on SES and breast cancer outcomes were screened and selected independently by two reviewers. Discrepancies were resolved by consensus or consultation with a third reviewer. Key SES indicators included income, education, and occupation, with breast cancer outcomes confirmed via receipt of treatment, survival rates, treatment adherence, and clinical outcomes.

### Data Extraction

The study characteristics, (author name, country, sample size), SES indicators (income, education, and occupation), and outcomes (survival, recurrence, or treatment receipt), were extracted independently by two reviewers using standardized Excel sheets. SES was categorized into quintiles, tertiles, or specific income and education levels where available. Risk of bias assessment was performed using the New Castle Ottawa scale (NOS) for observational studies.^19^ Two reviewers (N.Z. and J.A.) independently extracted the data and performed the quality appraisal. Any disagreements were resolved through the third reviewer (S.A.).

### Data Synthesis

Findings of the review are qualitatively summarized and presented the results in a tabular form. Due to the heterogeneity across study designs, SES measures, and outcome definitions, a meta-analysis was not feasible.

## Results

### Overview of Studies

**Figure 1.**
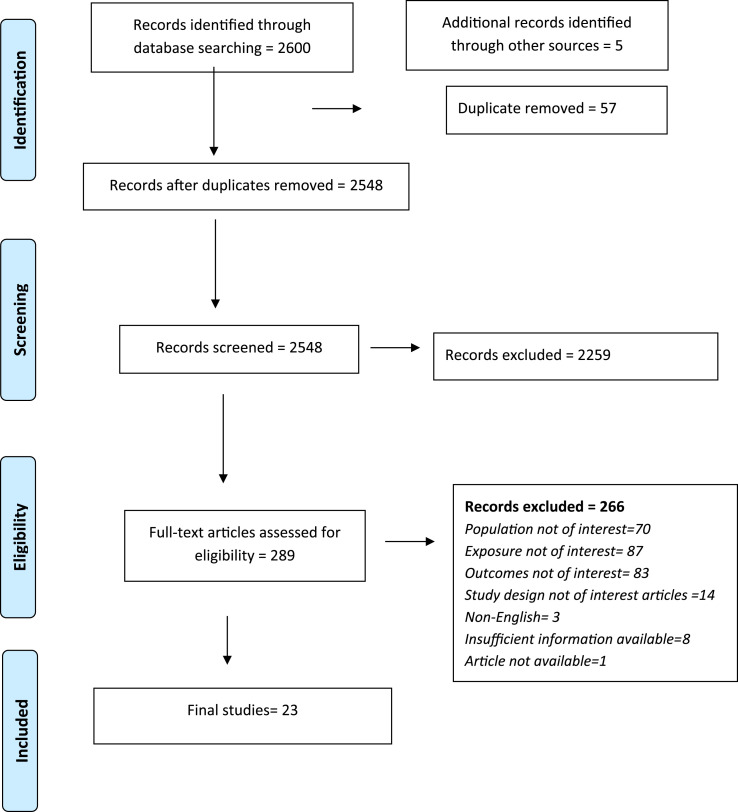
PRISMA Flow Diagram Showing Study Selection.

The search yielded a total of 2600 citations. Five additional studies were identified through bibliography of relevant studies. After removal of 57 duplicates, 2548 articles were screened based on the title and abstract. Of these, 2259 studies did not meet the inclusion criteria. The full-texts of the remaining 289 articles were assessed for eligibility. Of these, 266 did not meet the inclusion criteria. Overall, 23 studies met the inclusion criteria and were included in the review (Figure 1).

### Impact of Key SES Indicators on Breast Cancer Outcomes

A total of 23 (19 cohort; 4 cross-sectional) studies were included in this systematic review (Table 1), examining the association between income, education, and occupation as key SES indicators and breast cancer outcomes such as receipt of treatment, treatment adherence, survival, and access to care (Supplemental Table 1). In the included studies, results were reported using hazard ratios (HR), odds ratios (OR), and relative risks (RR), highlighting SES-based disparities.

**Table 1.** Study Characteristics of the Included Studies.

First author year	Study design	Sample size	Data source	Study period	Treatment received
Alderman et al., 2011^52^	Retrospective cohort	20 032	MEDPAR (Medicare Provider analysis and review), The national Medicare database	2003-2004	Breast conserving therapy
Ali et al., 2014^32^	Retrospective cohort	33 706	Florida cancer data system	1997-2002	BCS and RT
Bhutiani et al., 2020^21^	Retrospective cohort	6225	Kentucky cancer registry	2010-2016	Surgery, CT, RT
Fappiano et al, 2021^20^	Retrospective cohort	1 192 294	National cancer database	2004-2016	Surgery, CT, RT, or HT
Buszek et al., 2019^53^	Retrospective cohort	2995	National cancer database	2010-2014	Surgery, RT, ET
Byun et al., 2016^31^	Retrospective cohort	216 558	National cancer database	2004-2023	BCS, mastectomy (uni- and bilateral)
Caprio et al., 2012^54^	Retrospective cohort	387	National health interview survey	2010	No treatment
Chervu et al., 2023^22^	Retrospective cohort	1 079 057	National cancer database	2004-2015	BCS, mastectomy
Cheung et al, 2013^29^	Retrospective cohort	34 671	SEER, Georgia	2004-2009	RT
Dankwa-Mullan et al., 2021^23^	Cross-sectional	53 060	IBM MarketScan commercial claims and encounter and Medicare supplemental databases. Area health resource files provided non-clinical characteristics and sociodemographic data	2012-2018	BCS, mastectomy
Dreyer et al., 2018^17^	Retrospective cohort	11 368	SEER	2006-2009	Surgery, CT, RT, ET
Hedin et al., 2011^26^	Retrospective cohort	1402	Medicare	2005-2008	Surgery, HT
Jagsi et al., 2010^30^	Cross-sectional	2260	SEER	2005-2007	BCS, mastectomy, RT
Kong et al., 2018^33^	Retrospective cohort	27 706	SEER	2006-2009	Surgery, RT
McDougall et al, 2011^27^	Retrospective cohort	602	Cancer surveillance system of Western Washington	2004-2010	Adjuvant ET
Loghlin et al, 2016^24^	Retrospective cohort	581 514	National cancer database	1998-2012	BC treatment
Michalski et al, 1997^25^	Retrospective cohort	41 937	Medicare	1990	BCS, mastectomy
Neuner et al., 2019^28^	Cross-sectional	331	Greater plains collaborative sites (of a total of nine that treated adult breast cancer patients) from seven Midwestern states	2013-2014	Neoadjuvant, adjuvant CT
Patel et al., 2019^35^	Retrospective cohort	275	Publica and private hospitals (The private hospital is the flagship facility of an integrated academic medical center. The public hospital is a tertiary urban safety net hospital)	2011-2016	Surgery, neoadjuvant/adjuvant therapy
Sariego et al, 2015^34^	Retrospective cohort	537 059	American college of surgeons national cancer database benchmark reports	2000-2011	Mastectomy, BCS
Sura et al., 2015^55^	Retrospective cohort	8620	SEER	2000-2011	RT, BCT
Haji-Hersi et al., 2022^56^	Cross-sectional	32	Patients from non-randomized prospective study	2010-2017	ET
Griggs et al., 2007^57^	Prospective cohort	764	Awareness of neutropenia in cancer (ANC) study group registry	2002-2005	CT

Abbreviations: BC, Breast Cancer; BCS, Breast Conservation Therapy; CT, Chemotherapy; ET, Endocrine Therapy; HT, Hormone Therapy; RT, Radiotherapy.

#### Income

Nineteen studies reported income as one of the key SES indicators in determining treatment receipt and survival outcomes in breast cancer. Fappiano *et al* analyzed a large cohort of 1 192 294 women and reported that women with an income below $30,000 had a significantly higher likelihood of not receiving recommended surgical treatment, compared to higher-income patients (for < $30,000 vs ≥ $46,000, HR = 1.14, 95% confidence interval (CI): 1.06-1.22, *P* < 0.001).^20^ Another study by Bhutiani *et al* demonstrated that lower-income status was associated with delayed initiation of chemotherapy, which negatively impacted survival rates.^21^ Chervu *et al* highlighted that women in the lowest [0-25^th^ quartile] (adjusted odds ratio (aOR) = 0.95; 95% CI: 0.94-0.97) and second lowest [26^th^-50^th^ quartile] (adjusted OR = 0.98; 95% CI: 0.97-1.00) income quartiles were less likely to receive breast-conserving surgery (BCS) compared to those in the highest income quartile.^22^ This study emphasized that income constraints limited options for less invasive treatments, impacting patient’s quality of life.

A study by Danka-Mullan *et al* showed that communities with a higher median household income (per $10,000) were more likely to receive BCS (OR = 1.04; 95% CI: 1.00- 1.09; *P* = .04), suggesting income as a strong predictor of treatment type.^23^ Loghlin *et al* studied the demographic factors associated with poor cancer outcomes in the rural communities.^24^ The analysis showed that breast cancer patients from rural areas in the Southeast of US with lower median household incomes had shorter survival times compared to those with higher incomes.^24^ Specifically, patients with a median household income below $30,000 had a median survival of 147.98 months, while those with an income over $46,000 had a median survival of 176.1 months.^24^ Delays in initiating and completing treatment were more pronounced in low-income groups, contributing to a higher mortality risk. In another study by Michalski *et al* women in the wealthiest quartile (≥$45000) were approximately twice as likely to undergo BCS as those in the lowest income quartile (<$30,000), indicating that higher income is associated with access to preferred, less invasive treatment options.^25^

Hedin *et al* reported that among the 1402 breast cancer survivors included, 325 (23%) discontinued their hormonal therapy (HT) within 5 years of their surgery. Among the 280 individuals who provided reasons for early discontinuation, household income was a significant factor contributing to non-persistence (*P* < 0.05).^26^ McDougall *et al* analyzed a cohort of young breast cancer patients and reported that women with income under $50,000 were 2.4 times more likely to discontinue adjuvant HT than those with higher incomes, with women in the wealthiest quartile twice as likely to persist in treatment. The income disparity in the likelihood of discontinuing adjuvant HT is particularly distinct when comparing women with an income of ≥$90,000 to those with an income of <$25,000 (OR = 4.66; 95% CI: 1.91-11.36).^27^ Another study by Neuner *et al* showed that lower-income patients (<$100,000) had significantly lower odds of receiving neoadjuvant chemotherapy (adjusted OR = 0.56, 95% CI: 0.2-0.9).^28^

These findings demonstrate that lower-income status is associated with reduced access to BCS, higher likelihood of treatment discontinuation, and delayed treatment, all of which contribute to poorer survival outcomes among breast cancer patients.

#### Education

Sixteen studies reported education as one of the key SES indicators in breast cancer outcomes. Bhutiani *et al.* reported that lower education level was associated with delay in post operative chemotherapy (OR = 1.324; 95% CI: 1.164-1.506, *P* < .001).^21^ In a study by Cheung *et al* patients from counties with lower educational attainment had a 2% reduction in five-year survival probability compared to counties with at least 25% college graduates (*P* = .030).^29^ This study highlights that community-level education influenced survival, potentially due to varying levels of health literacy and access to information about treatment. In another study by Jagsi *et al* women without a college degree were significantly less likely to receive radiotherapy (RT; OR = 0.85; 95% CI: 0.78-0.92).^30^

A study by Byun *et al.* reported that women from highly educated areas were more likely to choose BCS over mastectomy, with patients in areas with a high percentage of college graduates showing a 1.23 times higher likelihood of selecting BCS (*P* < .001).^31^ Similarly, Ali *et al.* demonstrated that higher educational attainment was positively associated with choosing BCS, indicating that educated patients may have more resources and information available to make treatment choices.^32^

#### Occupation and other SES Factors

Lower occupational status, often linked to job inflexibility and limited medical leave, posed significant barriers to care. Kong *et al* examined occupational class as an SES indicator and demonstrated that patients in lower occupational categories (blue-collar workers) were less likely to receive chemotherapy or surgery, largely due to limited access to medical leave or job flexibility.^33^ Blue-collar workers had a 1.37 times higher risk of delayed treatment than white-collar workers (*P* < .01), highlighting occupational constraints as a barrier to timely care.^33^ A similar study by Sariego *et al* demonstrated the cumulative effects of income, education, and occupation on treatment outcomes, showing that women from lower-income, less-educated backgrounds with blue-collar jobs had lower rates of BCS (*P* < .001) and longer delays in initiating both chemotherapy and RT.^34^

Dreyer *et al* reported that patients from physically demanding jobs had a 25% higher risk of discontinuing endocrine therapy within the first year compared to those in less physically taxing occupations (OR = 1.25; 95% CI: 1.10-1.42).^17^ Another study by Patel *et al* focused on occupational disparities in survival rates, noting that patients in lower-status jobs without employer-based health insurance had significantly lower survival.^35^

Dankwa-Mullan *et al* examined multiple SES indicators and indicated that lower SES was consistently linked to longer delays in treatment and lower adherence to BCS.^23^ The study reinforced that SES constraints influence the timing and quality of breast cancer care. These cumulative SES affects often resulted in increased recurrence and poorer survival, underscoring the multifaceted role of SES in determining breast cancer outcomes.

## Discussion

The results of this systematic review highlight the influence of SES on breast cancer outcomes, emphasizing that key SES indicators income, education, and occupation collectively impact access to care, treatment adherence, and survival rates. These socioeconomic factors were consistently linked to disparities in treatment affordability, accessibility, quality, and patient survival rates, as highlighted in the literature on health inequities and social determinants of health.

To the best of our knowledge, this is the first systematic review to specifically examine the relationship between SES and breast cancer treatment in the US. A recent umbrella review by Li *et al* that summarized existing meta-analyses on SES and various cancer-related outcomes, showed that individuals with lower SES are disproportionately disadvantaged by reduced access to screening, access to immunotherapy, and targeted treatments across several cancer types. However, the review did not focus on treatment outcomes for breast cancer patients in particular, nor studies specifically from the US.^14^ In our study, income was found to be a significant determinant, with low income associated with decreased treatment adherence, reduced likelihood of receiving recommended therapies, and increased treatment discontinuation. A review by Devaux *et al* reported that such income-based gaps in the treatment exists, especially in rural or less developed areas where monetary constraints compel patients to delay treatment and even limit their access to advanced therapeutic options.^36^ Furthermore, income-based disparities were evident in survival outcomes, with higher-income women showing improved survival rates as a result of access to comprehensive treatment and supportive care.^37^ These income-driven disparities in receipt of treatment and adherence are critical, as they contribute to survival rates among low-income women, demonstrating that income-based inequities persist despite improvements in cancer care.

Education was shown to have a substantial effect on breast cancer outcomes in terms of health literacy, compliance with treatment guidelines, and preventive strategies. Studies indicated that patients with higher educational attainment were more likely to engage in consistent follow-up, adhere to recommended treatment regimens, and seek timely medical treatment, thereby decreasing their likelihood of presenting at advanced stages of the disease.^38^ Recent findings suggest that health literacy is closely linked to the level of education, empowering educated patients to make informed healthcare decisions, access to preventive care, and seek additional medical support when needed.^39^ Similarly, communities with higher average educational levels often benefit from health services oriented towards prevention, early detection, and health promotion strategies.^40^ Conversely, individuals with lower educational attainment may struggle with treatment complexities, resulting in negative health outcomes.^40^ A study by Padilla-Ruiz *et al*, showed that lower educational attainment is typically associated with delayed treatment and lower survival rates due to limited knowledge of breast cancer symptoms and available treatment options.^41^

Occupational status also played a substantial role, as women in physically demanding or low-wage jobs faced challenges in maintaining treatment schedules due to inflexible work hours, limited medical leave, and inadequate employer support.^17,33^ People with lower occupational status, particularly those without access to employer-sponsored health insurance, were more likely to delay or discontinue essential treatments, including chemotherapy and endocrine therapy.^17,33^ In contrast, white-collar workers, particularly those with employer-sponsored health insurance, tend to experience improved health outcomes due to better access to quality healthcare services.^42^ These findings illustrate that occupational constraints and job inflexibility further exacerbate the risk of nonadherence and poor survival outcomes in lower SES groups.

Reducing SES-related disparities in breast cancer management is essential to improving public health. Given the consistent association between SES and adverse breast cancer outcomes, targeted interventions are necessary to mitigate these disparities.^43^ Policy reforms that expand access to affordable healthcare, provide financial support for out-of-pocket expenses, and increased workplace accommodations for individuals undergoing cancer treatment are essential for reducing SES-based inequities in breast cancer care.^43^ Enhancing educational outreach and health literacy initiatives could further empower patients with lower education levels to participate in preventive care and make informed treatment decisions.^38^

Effective health policies must prioritize access to care, particularly among low-income and underinsured populations, through initiatives such as subsidized or publicly supported breast cancer screening and treatment programs.^14,43^ Targeted policy interventions may include expanding health insurance coverage for low-income populations, implementing cost-reduction programs to minimize out-of-pocket expenses, and improving workplace policies to allow flexibility for patients undergoing treatment.^14,43^ These measures could help address systemic barriers and promote more equitable breast cancer care across socioeconomic groups. In addition, evidence suggests that community-level approaches can effectively mitigate these discrepancies. For example, the combination of community-driven initiatives coupled with accessible diagnostic services could enhance participation in campaigns targeted at low SES communities, promoting early detection and intervention.^44^ Additionally, addressing income-related barriers, such as financial support for low-income families to cover medical expenses, could effectively reduce SES disparities in breast cancer treatment outcomes, alleviating some of the financial burdens that often prevent low-income patients from accessing timely care.^5^

Future research should prioritize the development and evaluation of interventions tailored to meet the specific needs of breast cancer patients with lower SES. Recent studies highlight the potential of mobile health technologies to improve follow-up care and medication adherence within low-income and marginalized population.^45^ These technologies may help address communication gaps between patients and healthcare providers, facilitating real-time patient monitoring and support, thereby potentially enhancing treatment adherence and outcomes. Additionally, future studies should explore the effects of SES-specific interventions, such as income-based healthcare strategies, examining their influence on survival rates and quality of life among breast cancer patients.^11^ Considering the significant barriers related to healthcare insurance and high out-of-pocket costs in the US, research should also explore insurance-focused strategies, such as expanding Medicaid or implementing slide-scale subsidies, to improve access to timely diagnosis and care.^46^ Beyond income and occupational status, broader social determinants of health, including housing stability and neighborhood socioeconomic characteristics should be considered given their influence on cancer prognosis.^47^ A comprehensive understanding of SES-related determinants in breast cancer management could inform the design of policies and strategies aimed at reducing health disparities across diverse populations.

This systematic review offers critical insights into the multifaceted influence of SES on breast cancer outcomes, reinforcing the importance of SES indicators income, education, and occupation in shaping access to care, treatment adherence, and survival rates. A key strength of this review is its comprehensive examination of SES-related barriers to breast cancer management, providing actionable evidence for policymakers and health practitioners. By including diverse study designs (retrospective cohort, case-control, and cross-sectional), this review captures SES-related disparities across different populations. The qualitative synthesis approach effectively organizes findings in a structured manner, facilitating the identification of key associations between SES indicators and breast cancer outcomes, which may inform targeted interventions and health policy reforms aimed at reducing inequities.

However, certain limitations should be acknowledged. The review focused on three key indicators of SES income, education, and occupation while excluding other SES indicators such as healthcare insurance, housing, neighborhood characteristics. This approach was intentional to focus only on the key indicators of SES. While a broader approach was considered, we determined that such an approach would have substantially increased the number of included studies beyond what would allow for meaningful synthesis within our resource and time constraints. Although this review focused on key SES indicators, it is important to note that pre-existing health conditions, such as diabetes or hypertension, may also influence breast cancer treatment and survival. These comorbidities could mediate or worsen the effects of low SES by influencing treatment decisions, increasing side effect risks, or limiting access to care.^48,49^ While our included studies did not consistently report on comorbidities, future research should examine how these health conditions interact with SES and can impact treatment outcomes. Although this review did not specifically include variables such as familial or social support, these factors likely play an important role in mediating the relationship between SES and breast cancer treatment outcomes. Prior studies suggest that strong social support networks can enhance treatment adherence and facilitate access to care.^50^ In addition, while this review discusses differences in treatment and outcomes among ethnic groups, more work is needed to understand how ethnicity and SES work together to influence care. For instance, language barriers, discrimination, and lack of trust in the healthcare system may make it challenging for women from some ethnic backgrounds to get timely and appropriate treatment.^51^ Another challenge of our review was the heterogeneity across studies regarding SES definitions and outcome measures, which limited the feasibility of conducting a meta-analysis and hindered data comparability. Variability in SES indicators such as different income thresholds or educational categorizations complicates generalizability across diverse patient populations. Additionally, the predominance of observational studies in the included literature restricts the ability to establish causal relationships between SES and breast cancer outcomes. While these studies provide insights on real-world patterns of care and disparities, these cannot definitively determine causality. Future research should incorporate more robust causal inference approaches such as quasi-experimental designs, natural experiments, or prospective cohort studies with detailed data collection to better understand the effects of SES on treatment decisions and outcomes. Furthermore, many studies lacked detailed adjustments for confounding variables, which may have impacted the robustness of associations reported. To address these limitations, future research should prioritize longitudinal designs with standardized SES and outcome definitions, which could enhance causal inference and offer more reliable insights into the effects of SES on breast cancer prognosis and survival outcomes.

In conclusion, this review emphasizes the significant impact of SES on breast cancer outcomes, with income, education, and occupation each serving as critical barriers to equitable care. Addressing these SES-driven disparities requires multifaceted policy solutions and tailored interventions to improve access to quality care and support for underserved populations, aiming to reduce mortality and enhance survivorship among women from lower SES backgrounds.

## Supplemental Material

Socioeconomic Status and Breast Cancer Treatment in the United States: Results From a Systematic Literature Review Supplemental Material for Socioeconomic Status and Breast Cancer Treatment in the United States: Results From a Systematic Literature Review by Sreelatha Akkala, Mohammed Zuber, Julie Atta, Nompumelelo O. Mzizi, and Akkula Jyothi in Cancer Control

## Data Availability

All data analyzed during this study are included in this published article.*

## Appendix

### Abbreviations

AACR: American Association for Cancer Research
AJCC: American Joint Committee on Cancer
APBI: Accelerated Partial Breast Irradiation
ASCO: American Society of Clinical Oncology
BCS: Breast conserving surgery
BCT: Breast Conservation Therapy
BMI: Body mass index
CD: Charlson/Deyo
CI: Confidence interval
DCIS: Ductal carcinoma in situ
ER: Estrogen receptor
ET: Endocrine therapy
HER-2: Human Epidermal Growth Factor Receptor-2
HR: Hazard ratio
HT: Hormonal therapy
MARS: Medication adherence rating scale
NCDB: National Cancer Database
OR: Odds ratio
OS: Overall survival
PR: Progesterone Receptor
PRISMA: Preferred Reporting Items for Systematic Reviews and Meta‐Analyses (PRISMA)
RR: Relative risk
RT: Radiotherapy
SEER: Surveillance Epidemiology and End Results Program
SES: Socioeconomic status
SLNB: Sentinel Lymph Node Biopsy
TTS: Time to surgery
US: United States
