# Supplementary material for: Socioeconomic Status and Breast Cancer Treatment in the United States: Results From a Systematic Literature Review
Source: Cancer Control. 2025 May 9;32:10732748251341520. doi: 10.1177/10732748251341520 (PMC12064903; doi:10.1177/10732748251341520)
Supplement: Socioeconomic Status and Breast Cancer Treatment in the United States: Results From a Systematic Literature Review [file sj-pdf-1-ccx-10.1177_10732748251341520.pdf]

Supplemental Table 1: Methods and results of included studies

	Study design and methods/Patient characteristics								Result	
**First author year**	**Study design; overall sample size**	**Treatment received Female, n (%)**	**Population description**	**Socioeconomic status indicators**	**Other explanatory/ predictors**	**Outcome measure/s**	**Participants' age Follow-up duration**	**Adjusted for**	**SES specific outcomes/other outcomes**	**Interpretation of result**
Alderman *et al.*, 2011 [1]	Retrospective cohort study; N=20,032	Receipt of BCT 20,032 (81.8%)	Female Medicare beneficiaries who were 68 years of age.<br>Incident breast cancer cases with or without RT and axillary lymph node dissection.	A summary measure of SES for each US Zip code using data on income, education, and occupation from the 2000 US Census.<br>A summary SES score was then grouped into quintiles ranging from 1 (lowest) to 5 (highest)	Patient socio-demographic characteristics (age, race, composite SES value, US state), clinical factors (Charlson score), surgeon characteristics (age and sex)	Receipt of BCT<br>Prevalence	Age, mean, years: 68<br>NA	patient-level factors (age, race, comorbidity score, and SES) and surgeon-level factors (age and sex)	Only 78.2% of patients in the lowest SES quintile received BCT, compared with 86.3% of those in the highest quintile (OR = 0.60; 95% CI: 0.52-0.68).<br>Advanced patient age (>85 vs <70 years (OR = 0.50; 95% CI: 0.42-0.59); comorbidities (>3 vs ≤3: (OR = 0.26; 95% CI: 0.24-0.28) were associated with low use of BCT	Elderly patients who were more socioeconomically disadvantaged were significantly less likely to receive BCT

	Study design and methods/Patient characteristics								Result	
**First author year**	**Study design; overall sample size**	**Treatment received Female, n (%)**	**Population description**	**Socioeconomic status indicators**	**Other explanatory/ predictors**	**Outcome measure/s**	**Participants' age Follow-up duration**	**Adjusted for**	**SES specific outcomes/other outcomes**	**Interpretation of result**
Bhutiani *et al.*, 2020 [2]	Retrospective cohort study; N = 6225	Receipt of up-front surgery	Adult women ≥20 years of age diagnosed with breast cancer in Kentucky, between 2010 and 2016.	Education level (high school graduation rate); Poverty level (2008-2012 American Community Survey data)	Patient sociodemographic characteristics (age, race, region of residence), tumor characteristics (tumor size, grade, stage), and treatment characteristics (up-front surgery type, reconstruction, academic center)	Delays in breast cancer treatment (surgery, chemotherapy, radiation)	Age <50: 1664; 50–64: 2965; 65–74: 1306; 75+: 290	Adjusted for patient, tumor, and treatment characteristics	Lower education level and higher poverty level were associated with delays in receiving chemotherapy (OR = 1.324, 95% CI: 1.164-1.506 for low education)	Patient, tumor, and socioeconomic factors influence timing of treatment in breast cancer care including delays in surgery and radiation

	Study design and methods/Patient characteristics								Result	
**First author year**	**Study design; overall sample size**	**Treatment received Female, n (%)**	**Population description**	**Socioeconomic status indicators**	**Other explanatory/ predictors**	**Outcome measure/s**	**Participants' age Follow-up duration**	**Adjusted for**	**SES specific outcomes/other outcomes**	**Interpretation of result**
Fappiano *et al.*, 2020 [3]	Retrospective cohort study; N = 1,192,294	Receipt of non-surgical treatment	Female patients aged >18 years treated for breast cancer between 2004–2016, excluding those with metastatic or recurrent disease (US NCDB).	Income (<$30k, $30-34k, $35-45k, ≥$46k), education (high school degree level), insurance status	Patient demographics (age, race), tumor characteristics (subtype, stage), treatment facility type (academic, community), region, comorbidities (Charlson score)	OS and barriers to surgery	Age >50: 74.3%; Age ≤50: 25.7%	Adjusted for patient demographics, tumor stage, treatment, and comorbidities	Lower income and lower education were associated with a higher likelihood of not receiving surgery (income < $30k, HR = 1.14, 95% CI: 1.06–1.22); higher Charlson score also associated with reduced likelihood of surgery	Socioeconomic disparities, particularly lower income, lower education, and being uninsured or having public insurance, were significant barriers to surgery. The study highlights how these factors influence treatment decisions and OS.

	Study design and methods/Patient characteristics								Result	
**First author year**	**Study design; overall sample size**	**Treatment received Female, n (%)**	**Population description**	**Socioeconomic status indicators**	**Other explanatory/ predictors**	**Outcome measure/s**	**Participants' age Follow-up duration**	**Adjusted for**	**SES specific outcomes/other outcomes**	**Interpretation of result**
Buszek *et al.*, 2019 [4]	Retrospective cohort study; N = 2,995	Adjuvant ET alone: 1,957 (65%) Adjuvant RT alone: 1,038 (35%)	Patients ≥70 years of age with T1N0 hormone receptor-positive, HER-2 negative breast cancer, treated with lumpectomy.	Income, education level (based on geographical location)	CD score, clinical stage, geographical location, ethnicity, treatment type	OS	Median age 78 years (range 70-90); Median follow-up of 45 months	Age, CD score, geographical location, treatment type, ethnicity	Older age and a CD score of 1 were associated with worse survival; urban areas compared to metropolitan areas were associated with improved survival.	Healthy older patients with lumpectomy followed by either adjuvant RT or ET have equivalent 5-year survival outcomes. Non-compliance with ET suggests potential for de-escalation of treatment.

	Study design and methods/Patient characteristics								Result	
**First author year**	**Study design; overall sample size**	**Treatment received Female, n (%)**	**Population description**	**Socioeconomic status indicators**	**Other explanatory/ predictors**	**Outcome measure/s**	**Participants' age Follow-up duration**	**Adjusted for**	**SES specific outcomes/other outcomes**	**Interpretation of result**
Ali *et al.*, 2014 [5]	Retrospective cohort study; N = 33,706	BCS with RT: 15,278 (58.62%); Mastectomy: 13,002 (38.61%)	Women aged >40 years diagnosed with localized breast cancer in Florida from 1997 to 2002	Linked to 2000 census data for education, income, and poverty at the census tract level	Patient demographic characteristics (age, race/ethnicity, marital status), health insurance type, tumor size, year of diagnosis, socioeconomic factors (poverty and education at the census tract level), and community factors	Receipt of BCS with RT	Age, mean: 66 years	Age, race/ethnicity, marital status, tumor size, insurance type, socioeconomic factors (poverty, education)	Medicare-insured women were more likely to receive BCS with RT (OR = 1.10; 95% CI: 1.02-1.18) than privately insured. Non-Hispanic Black Medicaid-insured women had higher odds of receiving RT than non-Hispanic Whites (OR = 2.08; 95% CI: 1.13-3.83). Uninsured and Medicaid-insured women had the lowest rates of receiving RT after BCS.	Women with private insurance, higher education, and smaller tumors were more likely to receive BCS with RT. Poverty and race/ethnicity were significant only in Medicaid-insured women, with Black women being more likely to receive RT than White women.

	Study design and methods/Patient characteristics								Result	
**First author year**	**Study design; overall sample size**	**Treatment received Female, n (%)**	**Population description**	**Socioeconomic status indicators**	**Other explanatory/ predictors**	**Outcome measure/s**	**Participants' age Follow-up duration**	**Adjusted for**	**SES specific outcomes/other outcomes**	**Interpretation of result**
Byun *et al*., 2016 [6]	Retrospective cohort study; N = 216,558	BCS: 71.7% Unilateral Mastectomy: 19.6% Bilateral Mastectomy: 8.3%	Women with DCIS breast cancer who underwent surgery between 2004 and 2013, identified in the NCDB.	Income, education	Facility location, rural population community, age, CD comorbidity index, DCIS subtype, tumor size, hormone receptor status	Surgical management of DCIS	Range not specified; increasing trend in bilateral mastectomy from 2004 to 2013	Demographic, socioeconomic, and clinicopathologic factors	Increasing trend in bilateral mastectomies; associated with recent diagnosis year, facility type, location, age, race, education, and tumor size ($p<0.01$)	Demographic and socioeconomic factors play a significant role in surgical decisions for DCIS.

	Study design and methods/Patient characteristics								Result	
**First author year**	**Study design; overall sample size**	**Treatment received Female, n (%)**	**Population description**	**Socioeconomic status indicators**	**Other explanatory/ predictors**	**Outcome measure/s**	**Participants' age Follow-up duration**	**Adjusted for**	**SES specific outcomes/other outcomes**	**Interpretation of result**
Caprio *et al.*, 2010 [7]	Cross-sectional survey; N = 387, representing 2,764,854 individuals	17 (3.95%) received no treatment for breast cancer	Patients surveyed in the National Health Interview Survey with a history of breast cancer	Insurance status, education, region of residence	Perceived risk of breast cancer	Lack of treatment for breast cancer	NA; 2010 NHIS data release	Age, insurance status, perceived risk	Factors associated with receiving no treatment included education (p = 0.003), insurance status (p = 0.009), and region of residence (p = 0.031). Insurance status (p = 0.001), perceived risk (p = 0.002), age (p = 0.024) were significantly associated with receipt of treatment	Nearly 4% of breast cancer patients did not receive any treatment. Young patients and those without private insurance are particularly vulnerable populations

	Study design and methods/Patient characteristics								Result	
**First author year**	**Study design; overall sample size**	**Treatment received Female, n (%)**	**Population description**	**Socioeconomic status indicators**	**Other explanatory/ predictors**	**Outcome measure/s**	**Participants' age Follow-up duration**	**Adjusted for**	**SES specific outcomes/other outcomes**	**Interpretation of result**
Chervu *et al.*, 2023 [8]	Retrospective cohort study; N = 1,079,057	BCT: 619,442 (57.4%), Mastectomy: 459,615 (42.6%)	Women undergoing elective mastectomy or BCT for early-stage breast cancer (2004-2015 NCDB dataset)	Income (quartiles), insurance (Medicare, Medicaid)	Race (White, Black, AAPI), hospital type (Academic, Community), age, tumor size, care at rural vs. urban hospitals	Use of BCT vs. mastectomy for early-stage breast cancer	Median age: BCT = 61<br>Mastectomy = 59	Age, race, income, insurance status, hospital characteristics, tumor size	Lower-income quartiles, Medicare/Medicaid coverage, associated with reduced BCT.<br>Income (Quartiles):<br>76th-100th Quartile: 37.2% BCT vs. 33.9% mastectomy (Ref)<br>51st-75th Quartile: Adjusted OR = 0.98 (95% CI: 0.97-1.00), p < .001<br>26th-50th Quartile: Adjusted OR = 0.95 (95% CI: 0.94-0.97), p < .001<br>0-25th Quartile: Adjusted OR = 0.95 (95% CI: 0.94-0.97), p < .001<br>**Insurance**:<br>Private: 56.2% BCT vs. 55.6% mastectomy (Ref)<br>Medicare: Adjusted	Being in the lowest 2 quartiles of income was associated reduced odds of undergoing BCT. Persistent racial and socioeconomic disparities were observed in receipt of BCT.

	Study design and methods/Patient characteristics								Result	
**First author year**	**Study design; overall sample size**	**Treatment received Female, n (%)**	**Population description**	**Socioeconomic status indicators**	**Other explanatory/ predictors**	**Outcome measure/s**	**Participants' age Follow-up duration**	**Adjusted for**	**SES specific outcomes/other outcomes**	**Interpretation of result**
Cheung *et al.*, 2013 [9]	Retrospective cohort study; N = 34,671	NA (focus on cause-specific survival)	Women with breast cancer cases from SEER Georgia registry (2004-2009)	Race (African American, other), education attainment	AJCC stage, ER/PR status, radiotherapy receipt, county college graduate percentage	Cause-specific breast cancer survival	2004-2009 (5-year follow-up)	AJCC stage, ER/PR status, race, education attainment of county of residence	Patients living in less educated (n = 19764) areas had statistically worse survival outcome (Kolmogorov Smirnov 2-sample test of college education attainment of county of residence: h = 1; p = 0.0300; k = 0.2429). 2% worse survival for counties with lower education (≤25% college graduates) at 5 years<br>Lack of radiotherapy receipt higher in less educated counties (56%) compared to higher educated counties (46%)	Socioeconomic factors (race, education attainment of county) significantly influence survival outcomes in breast cancer patients, with lower survival for African American women and women in less educated counties.

	Study design and methods/Patient characteristics								Result	
**First author year**	**Study design; overall sample size**	**Treatment received Female, n (%)**	**Population description**	**Socioeconomic status indicators**	**Other explanatory/ predictors**	**Outcome measure/s**	**Participants' age Follow-up duration**	**Adjusted for**	**SES specific outcomes/other outcomes**	**Interpretation of result**
Dankwa-Mullan *et al.*, 2021 [10]	Cross-sectional retrospective study; N = 53,060	BCS: 68.4% (n = 36,270), Mastectomy: 31.6% (n = 16,790)	Women with non-metastatic invasive breast cancer from IBM MarketScan databases (2012-2017)	Household income, percentage with 4-year college degree, urbanization level, community healthcare provider availability	Age, genetic test, adjuvant therapies, region of residence, health plan type, availability of medical genetics, ob-gyn, and plastic surgeons	Type of surgical treatment (BCS vs. mastectomy); TTS	Median age: BCS = 59.7, Mastectomy = 55.4; Follow-up duration: 6 months	Age, income, community health service availability, adjuvant therapies, comorbidities	**Household income**: OR = 1.04 (95% CI: 1.00-1.09), p = 0.04 for BCS **Education**: Higher education associated with longer TTS Urban residence linked to longer TTS for mastectomy (12.78 days longer) Communities with more ob-gyn and medical geneticists had higher BCS rates (OR = 5.88 for medical genetics, p = 0.02)	Socioeconomic factors (income, education, healthcare access) significantly affect both choice of BCS and timing of surgery. Older patients, higher-income areas, and regions with more healthcare providers are more likely to have BCS over mastectomy.

	Study design and methods/Patient characteristics								Result	
**First author year**	**Study design; overall sample size**	**Treatment received Female, n (%)**	**Population description**	**Socioeconomic status indicators**	**Other explanatory/ predictors**	**Outcome measure/s**	**Participants' age Follow-up duration**	**Adjusted for**	**SES specific outcomes/other outcomes**	**Interpretation of result**
Dreyer *et al.*, 2018 [11]	Retrospective cohort study; N = 11,368	Axillary surgery: 87%, SLNB only: 33%, ALND only: 17%, Radiation: 81%	Elderly women aged 66-90 with early-stage breast cancer (2006-2009 SEER-Medicare)	Medicaid enrollment, census tract poverty level (poor, near-poor, high SES)	Age, race, hormone receptor status, node status, urban/rural residence, SEER stage	Receipt of axillary surgery, radiation post-BCS, adjuvant/neoadjuvant chemotherapy, ET	Mean age: 76 (6 years); 2006-2009	Age, race, hormone receptor status, cancer stage, node status, urbanization, comorbidities	Poor and near-poor women were less likely than high SES women to receive sentinel lymph node biopsy and radiation after breast conserving surgery (BCS). Poor women were also less likely than near-poor or high SES women to receive any axillary surgery and adjuvant chemotherapy.<br>Poor SES: Less likely to receive axillary surgery (OR: 0.69, p < 0.01), SLNB (OR = 0.71, p < 0.01), radiation post-BCS (OR = 0.59, p < 0.01), adjuvant chemotherapy (OR = 0.74, p < 0.01)<br>Near-poor SES: Less likely to receive	Significant disparities exist in receipt of newer and less invasive treatments for breast cancer based on SES.

	Study design and methods/Patient characteristics								Result	
**First author year**	**Study design; overall sample size**	**Treatment received Female, n (%)**	**Population description**	**Socioeconomic status indicators**	**Other explanatory/ predictors**	**Outcome measure/s**	**Participants' age Follow-up duration**	**Adjusted for**	**SES specific outcomes/other outcomes**	**Interpretation of result**
Hedin *et al.*, 2011 [12]	Retrospective cohort study; N = 1,402	Persistence with adjuvant hormone therapy	Medicare breast cancer patients, aged >65, who underwent initial surgery for Stage 1 or 2 breast cancer, residing in California, Florida, New York, or Illinois, and initiated HT within 1 year of surgery.	Income, insurance status, and household income as collected through survey responses and Medicare records.	Age, marital status, household income, and supplemental insurance in addition to Medicare.	Persistence with adjuvant hormone therapy.	Age: > 65; Follow-up duration: 5 years	Age, marital status, household income, supplemental insurance, and reasons for non-persistence.	Of the 1,402 patients studied, 23% discontinued HT within 5 years. The most common reasons were side effects (47%), belief that therapy was complete (17%), physician-directed discontinuation (15%), and cost (8%).	Non-persistence in HT was common among older breast cancer survivors, with side effects and cost being significant factors. Persistence was influenced by demographic factors such as age, marital status, and income, with poorer insurance coverage contributing to higher non-persistence rates.

	Study design and methods/Patient characteristics								Result	
**First author year**	**Study design; overall sample size**	**Treatment received Female, n (%)**	**Population description**	**Socioeconomic status indicators**	**Other explanatory/ predictors**	**Outcome measure/s**	**Participants' age Follow-up duration**	**Adjusted for**	**SES specific outcomes/other outcomes**	**Interpretation of result**
Jagsi *et al.*, 2010 [13]	Survey-based study; N = 2,260	Receipt of adjuvant RT	Women aged 20-79 with nonmetastatic breast cancer, diagnosed between July 2005 and February 2007, living in Los Angeles and Detroit; SEER registries were used to identify the patients.	Income and insurance status based on survey responses and SEER data.	Surgery type (lumpectomy or mastectomy), patient preferences, surgeon involvement, comorbidities, age, indication strength (based on tumor stage and node status).	Receipt of adjuvant RT	Age range: 20-79 years; No specific follow-up duration	Surgery type, indication strength, comorbidities, age, income, ethnicity, insurance status, and surgeon involvement.	RT receipt was higher after lumpectomy (95.4% with strong indications) compared to mastectomy (77.6% with strong indications). Income, age, and surgeon participation were significant predictors of RT receipt. Patients undergoing mastectomy with strong indications for RT were less likely to receive RT if their surgeon's involvement was lower.	RT underutilization after mastectomy was notable, especially when provider involvement was low. Surgeon participation had a strong influence on RT receipt, even for patients expressing a desire to avoid RT. Interventions targeting both patient education and surgeon involvement may improve RT utilization after mastectomy.

	Study design and methods/Patient characteristics								Result	
**First author year**	**Study design; overall sample size**	**Treatment received Female, n (%)**	**Population description**	**Socioeconomic status indicators**	**Other explanatory/ predictors**	**Outcome measure/s**	**Participants' age Follow-up duration**	**Adjusted for**	**SES specific outcomes/other outcomes**	**Interpretation of result**
Kong *et al.*, 2018 [14]	Population-based cohort study; N = 27,706	Breast cancer treatments (e.g., surgery, chemotherapy, radiation)	Elderly women with incident breast cancer from SEER-Medicare database	SES measured by per capita income, poverty level, household income	Tumor characteristics (e.g., size, stage), patient demographics (age, race, comorbidities)	5-year survival	Age: 66-90, Follow-up: 5 years	Adjusted for age, comorbidities, treatment type, tumor characteristics	Survival probabilities by SES: low SES women were more likely to have poor outcomes	Women of lower SES had significantly lower survival rates compared to higher SES women due to differences in treatment patterns

	Study design and methods/Patient characteristics								Result	
**First author year**	**Study design; overall sample size**	**Treatment received Female, n (%)**	**Population description**	**Socioeconomic status indicators**	**Other explanatory/ predictors**	**Outcome measure/s**	**Participants' age Follow-up duration**	**Adjusted for**	**SES specific outcomes/other outcomes**	**Interpretation of result**
McDougall *et al.*, 2011 [15]	Cohort study; N = 602	Receipt of adjuvant HT	Women aged 20-45 with a primary diagnosis of invasive estrogen receptor-positive breast cancer between 2004-2010 in Western Washington	Income categories (<$50K, ≥$50K, <$25K, ≥$90K)	Demographic factors, breast cancer risk factors	Receipt and discontinuation of adjuvant HT (e.g., Tamoxifen, Letrozole)	Age 20-45, Follow-up: time of interview	Age, household size, income level	Women with income <$50K had 2.4 times higher odds of discontinuing adjuvant HT compared to those with ≥$50K; higher income associated with better adherence	Women with lower income were more likely to discontinue adjuvant HT, indicating financial constraints may affect adherence and outcomes.

	Study design and methods/Patient characteristics								Result	
**First author year**	**Study design; overall sample size**	**Treatment received Female, n (%)**	**Population description**	**Socioeconomic status indicators**	**Other explanatory/ predictors**	**Outcome measure/s**	**Participants' age Follow-up duration**	**Adjusted for**	**SES specific outcomes/other outcomes**	**Interpretation of result**
Loughlin *et al.*, 2016 [16]	Retrospective cohort study; N = 15,000	Various breast cancer treatment	Adult women with breast cancer from a national health database	Income levels, education level, residential area	Tumor stage, comorbidities, health insurance	Mortality, disease recurrence, treatment adherence	Mean age: 57 years, Follow-up: 5 years	Age, comorbidities, health insurance, tumor stage	Median survival of 147.98 months for income <$30,000; 176.1 months for income >$46,000; lower income linked to higher treatment delays and mortality risk	Low-income women experienced worse outcomes, highlighting the need for tailored interventions for disadvantaged groups.

First author year	Study design and methods/Patient characteristics								Result	
	**Study design; overall sample size**	**Treatment received Female, n (%)**	**Population description**	**Socioeconomic status indicators**	**Other explanatory/ predictors**	**Outcome measure/s**	**Participants' age Follow-up duration**	**Adjusted for**	**SES specific outcomes/other outcomes**	**Interpretation of result**
Michalski *et al.*, 1997 [17]	Retrospective cohort study; N = 41,937	BCS: 15.1% (6,329) Mastectomy: 84.9% (35,608)	Female Medicare beneficiaries aged 65-79 with local or regional breast carcinoma treated in 1990	Income, education, poverty level, vacant housing (based on zip code)	Black race, tumor stage, urban vs. rural status	Likelihood of receiving BCS over mastectomy	Age 65-79; Follow-up: NA	Age, race, SES factors, urban/rural, stage	Women in the wealthiest quartile were approximately twice as likely to undergo BCS as those in the poorest quartile. Higher educational status was positively associated with receipt of BCS, and women in the highest quartile of educational status of the zip code were also approximately twice as likely to undergo BCS as women in the lowest quartile. Women are approximately 40% less likely to undergo BCS when living in areas with ≥10% of the population below the poverty level	Women from higher SES backgrounds were significantly more likely to receive BCS

	Study design and methods/Patient characteristics								Result	
**First author year**	**Study design; overall sample size**	**Treatment received Female, n (%)**	**Population description**	**Socioeconomic status indicators**	**Other explanatory/ predictors**	**Outcome measure/s**	**Participants' age Follow-up duration**	**Adjusted for**	**SES specific outcomes/other outcomes**	**Interpretation of result**
Neuner *et al.*, 2019 [18]	Multicenter survey study; N = 331	Neoadjuvant chemotherapy (29.6%) vs. adjuvant chemotherapy (70.4%)	Women diagnosed with Stage I-III breast cancer in 2013-2014 across 8 medical institutions in the US	Income (<$100,000 vs. >$100,000), education level	Tumor stage, HER2/hormone receptor status, urban vs. rural residence	Receipt of neoadjuvant chemotherapy	Age: 18+<br>No specified follow-up duration	Age, cancer stage, biomarker status	Lower-income women (<$100,000) had significantly lower odds of receiving neoadjuvant chemotherapy (adjusted OR = 0.56, 95% CI: 0.2–0.9)	Lower-income women were less likely to receive neoadjuvant chemotherapy, possibly contributing to treatment disparities in outcomes.

	Study design and methods/Patient characteristics								Result	
**First author year**	**Study design; overall sample size**	**Treatment received Female, n (%)**	**Population description**	**Socioeconomic status indicators**	**Other explanatory/ predictors**	**Outcome measure/s**	**Participants' age Follow-up duration**	**Adjusted for**	**SES specific outcomes/other outcomes**	**Interpretation of result**
Patel *et al.*, 2019 [19]	Retrospective cohort study; N = 275	Mastectomy: 170 (65.2%) Lumpectomy: 105 (34.8%)	Women under 45 years diagnosed with invasive breast cancer (Stage I-III) at BH and a PCC	Income (insurance status): 70% uninsured at BH vs. 20% at PCC Education level: 50% with < high school education at BH vs. 10% at PCC Language: 40% LEP at BH vs. 5% at PCC	Tumor stage, race/ethnicity, palpable mass	Type of surgical treatment received (mastectomy, lumpectomy, reconstruction)	Age <45; 2011-2016	Race, language, education, tumor stage	Lower education level, higher LEP at BH, and more advanced tumor stage at BH. Reconstruction rates: 25% at BH vs. 28% at PCC despite SES differences	Despite SES differences, both hospitals provided similar surgical treatment options. BH patients presented with more advanced cancer but SES did not significantly affect treatment choice.

	Study design and methods/Patient characteristics								Result	
**First author year**	**Study design; overall sample size**	**Treatment received Female, n (%)**	**Population description**	**Socioeconomic status indicators**	**Other explanatory/ predictors**	**Outcome measure/s**	**Participants' age Follow-up duration**	**Adjusted for**	**SES specific outcomes/other outcomes**	**Interpretation of result**
Sariego *et al.*, 2015 [20]	Retrospective cohort study; N = 537,059	BCS: 64.4% Mastectomy: 35.6%	Women with early-stage breast cancer (Stages 0, I, II) from American College of Surgeons NCDB (2000-2011)	Income categories: < \$28K (Group A), \$28K-\$49K (Group B), > \$49K (Group C); Race: Caucasian, non-Caucasian	Age, tumor stage, geographic location	BCS rates by SES and race	Not specified	Age, race, SES, tumor stage, geography	A difference with regard to socioeconomic status was observed. This relationship between household income and BCS rates was linear, direct, and statistically significant.<br>BCS by income: Group A - 62.6%, Group B - 63.7%, Group C - 65.9%	SES significantly influenced BCS rates, with higher rates among higher-income groups. Racial disparities in BCS rates were minor after adjusting for SES.

	Study design and methods/Patient characteristics								Result	
**First author year**	**Study design; overall sample size**	**Treatment received Female, n (%)**	**Population description**	**Socioeconomic status indicators**	**Other explanatory/ predictors**	**Outcome measure/s**	**Participants' age Follow-up duration**	**Adjusted for**	**SES specific outcomes/other outcomes**	**Interpretation of result**
Sura *et al.*, 2021 [21]	Retrospective cohort study; N = 8,620	RT: 75% (6,446) No RT: 25% (2,174)	Women aged ≥60 years with early-stage, low-risk breast cancer who underwent lumpectomy (2000-2011, SEER data)	Insurance status, income, education, marital status, race	Tumor size, lymph node involvement, ER/PR status	Receipt of RT as part of BCT	Age: ≥60; No specified follow-up duration	Age, marital status, race, income, education	**Income**: Higher income increased RT probability (median income \$73,770 with RT vs. \$72,830 without, p = 0.037) **Education**: High school graduates more likely to receive RT (p < 0.0001); Bachelor's degree increased APBI probability (p = 0.013)	Lower SES (income and education), race (Black), and marital status (unmarried/widowed) associated with lower RT receipt as part of BCT.

	Study design and methods/Patient characteristics								Result	
**First author year**	**Study design; overall sample size**	**Treatment received Female, n (%)**	**Population description**	**Socioeconomic status indicators**	**Other explanatory/ predictors**	**Outcome measure/s**	**Participants' age Follow-up duration**	**Adjusted for**	**SES specific outcomes/other outcomes**	**Interpretation of result**
Haji-Hersi *et al.*, 2022 [22]	Prospective survey study; N = 32	ET (e.g., Tamoxifen, Aromatase Inhibitors)	Women with ER-positive breast cancer from a single institution, surveyed between 2010-2017	Education level (high school, some college, college degree, professional degree), income level	Drug allergies, adverse events, patient beliefs, cost of treatment, patient-provider relationship	Adherence to endocrine therapy (measured by MARS-8 scale)	Average age: 58.8 years	Education, beliefs, income, adverse events	Low education level and higher out-of-pocket costs were significantly associated with non-adherence (p = 0.025 and p = 0.005, respectively); adverse drug events also increased non-adherence (p = 0.026)	Drug-related (allergies, adverse effects) and socioeconomic factors (education, cost) are key barriers to therapy adherence. Patient beliefs and the patient-provider relationship play an essential role in adherence.

	Study design and methods/Patient characteristics								Result	
**First author year**	**Study design; overall sample size**	**Treatment received Female, n (%)**	**Population description**	**Socioeconomic status indicators**	**Other explanatory/ predictors**	**Outcome measure/s**	**Participants' age Follow-up duration**	**Adjusted for**	**SES specific outcomes/other outcomes**	**Interpretation of result**
Griggs *et al.*, 2007 [23]	Prospective observational cohort study; N = 764	Adjuvant chemotherapy (dose reductions evaluated)	Women with early-stage (I-III) breast cancer treated with standard chemotherapy regimens across 91 sites in the US	Education level, median household income, poverty rate by zip code	BMI, geographic region, comorbidity index	First cycle chemotherapy dose reductions (below 85% of standard)	Mean age: 53.2 years	Age, BMI, tumor stage, insurance, education, region	Higher rates of dose reduction in patients with < high school education (32.7%), lower zip code income levels, and obesity (OR for severe obesity: 4.04)	Socioeconomic factors, especially low education, along with obesity, were linked to lower chemotherapy doses, highlighting disparities in treatment delivery.

AJCC: American Joint Committee on Cancer; APBI: Accelerated Partial Breast Irradiation; BCT: Breast Conservation Therapy; BH: Public safety net hospital; BMI: Body Mass Index; CI: Confidence Interval; CD: Charlson/Deyo; DCIS: Ductal Carcinoma In Situ; ER: Estrogen Receptor; ET: Endocrine Therapy; HER-2: Human Epidermal Growth Factor Receptor-2; HT: Hormone Therapy; IBM: International Business Machines; LEP: Limited English Proficiency; MARS: Medication Adherence Rating Scale; N: Sample size; NA: Not Applicable; NCDB: National Cancer Database; OR: Odds Ratio; OS: Overall Survival; PCC: Private Cancer Centre; PR: Progesterone Receptor; RT: Radiotherapy; SES: Socioeconomic Status; SEER: Surveillance Epidemiology and End Results Program; SLNB: Sentinel Lymph Node Biopsy; TTS: Time to surgery; US: United State
